# Variability of Inducible Expression across the Hematopoietic System of Tetracycline Transactivator Transgenic Mice

**DOI:** 10.1371/journal.pone.0054009

**Published:** 2013-01-11

**Authors:** Megumi Takiguchi, Lukas E. Dow, Julia E. Prier, Catherine L. Carmichael, Benjamin T. Kile, Stephen J. Turner, Scott W. Lowe, David C. S. Huang, Ross A. Dickins

**Affiliations:** 1 Molecular Medicine Division, Walter and Eliza Hall Institute of Medical Research, Parkville, Victoria, Australia; 2 Chemical Biology Division, Walter and Eliza Hall Institute of Medical Research, Parkville, Victoria, Australia; 3 Cancer and Haematology Division, Walter and Eliza Hall Institute of Medical Research, Parkville, Victoria, Australia; 4 Department of Medical Biology, University of Melbourne, Parkville, Victoria, Australia; 5 Department of Microbiology and Immunology, University of Melbourne, Parkville, Victoria, Australia; 6 Memorial Sloan-Kettering Cancer Center, New York, New York, United States of America; 7 Howard Hughes Medical Institute, New York, New York, United States of America; Maisonneuve-Rosemont Hospital, Canada

## Abstract

The tetracycline (tet)-regulated expression system allows for the inducible overexpression of protein-coding genes, or inducible gene knockdown based on expression of short hairpin RNAs (shRNAs). The system is widely used in mice, however it requires robust expression of a tet transactivator protein (tTA or rtTA) in the cell type of interest. Here we used an *in vivo* tet-regulated fluorescent reporter approach to characterise inducible gene/shRNA expression across a range of hematopoietic cell types of several commonly used transgenic tet transactivator mouse strains. We find that even in strains where the tet transactivator is expressed from a nominally ubiquitous promoter, the efficiency of tet-regulated expression can be highly variable between hematopoietic lineages and between differentiation stages within a lineage. In some cases tet-regulated reporter expression differs markedly between cells within a discrete, immunophenotypically defined population, suggesting mosaic transactivator expression. A recently developed CAG-rtTA3 transgenic mouse displays intense and efficient reporter expression in most blood cell types, establishing this strain as a highly effective tool for probing hematopoietic development and disease. These findings have important implications for interpreting tet-regulated hematopoietic phenotypes in mice, and identify mouse strains that provide optimal tet-regulated expression in particular hematopoietic progenitor cell types and mature blood lineages.

## Introduction

Genetically modified mice are important tools for the study of mammalian gene function *in vivo*. Targeted gene modification using homologous recombination in mouse embryonic stem cells allows production of mice with mutations in specific genes, and the Cre/lox system allows conditional deletion of genes in particular cell types [1]–[3]. The tetracycline (tet)-regulated expression system is also widely used in mouse models, with the key advantage of temporal and reversible control of transgene expression. Originally developed for inducible gene overexpression [4]–[6], it also allows inducible gene knockdown via expression of microRNA-based short hairpin RNAs (shRNAs) [7]–[9].

The tet-regulated system requires two genetic components: a tet response element (TRE) promoter controlling mRNA or shRNA expression, and a recombinant tet-transactivator that can activate the TRE promoter. The tTA (tet-off) transactivator binds and activates the TRE promoter but is inhibited by the administration of tetracycline or its commonly used analog doxycycline (Dox). Conversely, the rtTA (tet-on) transactivator is latent until activated by Dox. Therefore Dox indirectly controls expression from the TRE by reversibly regulating transactivator function.

*In vivo* tet-regulated protein or shRNA expression is commonly achieved by crossing mice carrying a TRE promoter cassette transgene with mice carrying a tet transactivator transgene, resulting in progeny carrying both genetic components. An important factor in effective tet-regulated expression is the genomic location of the TRE promoter cassette, which influences its accessibility by the tet transactivator. Hence, recent approaches have targeted the TRE cassette to defined genomic loci to optimise inducible expression in most cell types [9], [10]. A second key determinant of effective tet-regulation is the expression level of the tet transactivator. Many mouse strains have been generated that express the tTA or rtTA transactivators under the control of different promoters (www.tetsystems.com). Although many of these promoters are nominally ubiquitous or tissue-specific, in most cases the pattern and abundance of transactivator expression in these mouse strains is poorly characterised. In order to optimally utilise transgenic, tet-regulated expression systems in mice, and to rationally interpret the resulting phenotypes, an understanding of the strength and breadth of transactivator function in particular cell types *in vivo* is imperative. In this study we have examined *in vivo* transactivator function across the hematopoietic system of several commonly used transactivator mouse strains.

## Results

### Characterising Tet-regulated Expression in Hematopoietic Stem and Progenitor Cells

To examine tet-regulated expression in the hematopoietic system of transgenic transactivator mouse strains, we utilised a reporter mouse strain where expression of green fluorescent protein (GFP) is under the control of the TRE promoter. The 3′ UTR of the GFP-encoding transcript in this reporter strain also includes a microRNA-based shRNA targeting firefly luciferase (Luc.1309 or shLuc) [9]. We have previously used this TRE-GFP-shLuc strain as a negative control in tet-regulated shRNA studies [9], [11]. The TRE-GFP-shLuc transgene is targeted to the *collagen type I alpha* (*Col1a1*) locus, previously shown to facilitate tet-regulated expression in a wide range of cell types *in vivo* [9], [10]. GFP expression was negligible in hematopoietic cells of TRE-GFP-shLuc single transgenic mice, verifying that reporter expression is not leaky (Figure S1).

We crossed reporter mice to various transgenic transactivator mouse strains procured or produced by our laboratories. More recently developed tet-on mouse strains often express the M2-rtTA or rtTA3 transactivators, which have improved transcriptional activity and Dox-sensitivity relative to the original rtTA protein [12], [13]. Of the six transgenic transactivator mouse strains we examined, four express the tet-on transactivator: CAG-rtTA3 [9], CMV-rtTA [14], ROSA26-M2rtTA [15], and Vav-rtTA3. The CAG, CMV, and ROSA26 promoters are often regarded as ‘ubiquitous’ promoters and are widely used to drive a broad expression pattern in transgenic mice. The CAG promoter contains an enhancer element from human cytomegalovirus (CMV) together with sequences from the chicken ß-actin promoter and rabbit ß-globin genes that yield high level expression in mammalian cells [16]. Similarly, the CMV promoter is based on the strong promoter of the immediate early gene of human CMV [17]. The ROSA26 promoter was originally identified based on its broad expression pattern during mouse embryogenesis but is also widely active in adult tissues [18], [19]. In ROSA26-M2rtTA mice M2rtTA expression is driven by the endogenous ROSA26 promoter, whereas the other five strains tested were all originally generated by pronuclear injection of synthetic expression cassettes resulting in variable transgene insertion site and copy number. To complement the broadly acting tet-on mouse strains CAG-rtTA3, CMV-rtTA, and ROSA26-M2rtTA, we used standard pronuclear transgenesis to generate a transgenic mouse strain where expression of the rtTA3 transactivator is under control of the Vav promoter. Vav promoter activity is mainly restricted to the hematopoietic compartment of mice, where it drives expression across all blood cell types [20]. After screening several independent transgenic founder lines we identified one (hereafter referred to as Vav-rtTA3) that showed particularly effective tet-regulated reporter expression in blood cells (see below).

In addition to four tet-on mouse strains, we tested two previously described transgenic strains that express the tet-off transactivator from the well characterised hematopoietic promoters Vav and Eμ. In contrast to the four tet-on strains, which were all maintained on a C57BL/6 background, the two tet-off strains were FVB/N strain background. Vav-tTA mice were originally developed to drive tet-regulated oncogene expression across the hematopoietic system [21]. Eμ-tTA mice express tTA under the control of the immunoglobulin heavy chain (IgM) enhancer and the SRα promoter [22]. The Eμ enhancer is particularly active in developing B and T lymphocytes [23], and effectively drives lymphoid-specific expression in mouse models [24].

**Figure 1:**
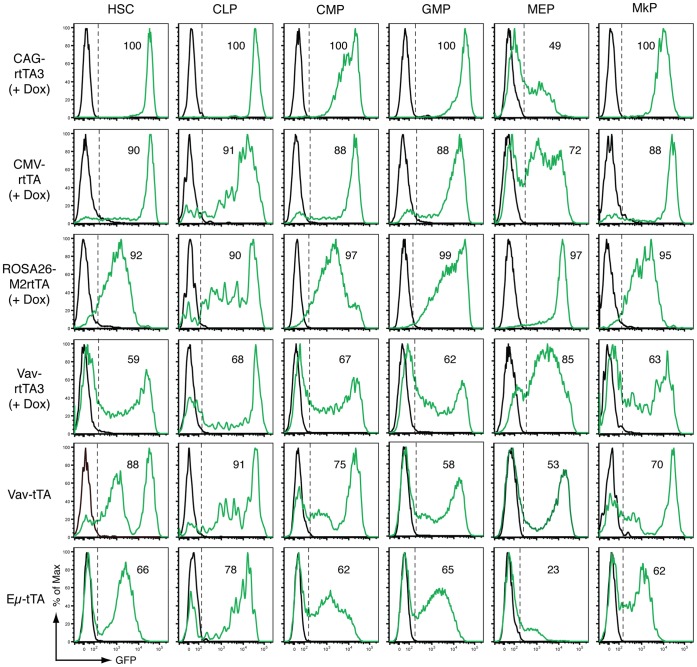
Tet-regulated GFP reporter expression in hematopoietic stem cells and early progenitors of tet-transactivator transgenic mice. Flow cytometry profiles of GFP expression in hematopoietic stem and progenitor cells isolated from the bone marrow of various transgenic mouse strains. Profiles from a representative mouse (n = 2 mice analysed per genotype) carrying the indicated transactivator transgene along with the TRE-GFP-shLuc reporter transgene are shown in green, with wild type controls shown in black. Tet-on bitransgenic reporter mice (CAG-rtTA3, CMV-rtTA, ROSA26-M2rtTA, Vav-rtTA3) were given Dox food for 7 days before analysis, whereas tet-off bitransgenic reporter mice (Eµ-tTA and Vav-tTA) were untreated. The percentage of GFP+ cells in each population is indicated. HSC: Lin–Sca1+Kit+ (LSK) hematopoietic stem cell. CLP: Lin–Kit^Int^Sca1+CD127+ common lymphoid progenitor. CMP: Lin–Sca1–Kit+CD34+FcγRII/III– common myeloid progenitor. GMP: Lin–Sca1–Kit+CD34+FcγRII/III+ granulocyte/macrophage progenitor. MEP: Lin–Sca1–Kit+CD34–FcγRII/III– megakaryocyte/erythroid progenitor. MkP: Lin–Sca1–Kit+CD41+CD150+ megakaryocyte progenitor. Gating strategies are shown in Figure S2.

In all cases adult mice carrying transactivator and reporter transgenes developed normally and were analysed alongside littermate single transgenic or wild type controls. For tet-on strains, administering Dox food for one week prior to analysis activated reporter expression. GFP expression was determined in various hematopoietic cell types using flow cytometry of cells isolated from bone marrow, spleen, thymus and blood. We initially focused on stem cells and early progenitors of the lymphoid and myeloid lineages. Using well-defined surface antigens to identify cell types (Figure S2), we examined tet-regulated GFP reporter expression in populations of cells enriched for hematopoietic stem cells (HSCs), common myeloid progenitors (CMPs), granulocyte/macrophage progenitors (GMPs), megakaryocyte/erythroid progenitors (MEPs) and megakaryocyte progenitors (MkPs) (Figure 1). Notably, each transactivator strain showed a distinct GFP expression profile across these cell types. Of all the transactivator strains analysed, CAG-rtTA3 consistently drove highly efficient (near 100%) and intense GFP expression in HSCs and early progenitors, with the exception of MEPs (Figure 1). GFP expression driven by CMV-rtTA was similar to CAG-rtTA3 but less uniformly intense, and approximately 10% of most progenitor cell populations in this strain failed to express GFP. The ROSA26-M2rtTA strain showed more heterogeneous GFP induction between and within different stem and progenitor populations, with poor expression intensity in HSCs compared with the CAG-rtTA3 and CMV-rtTA strains. However ROSA26-M2rtTA drove uniformly high reporter expression in MEPs, a cell type with weak or heterogeneous reporter expression in the other five strains examined (Figure 1). In keeping with the previously described pan-hematopoietic expression pattern of the Vav promoter [20], we observed reporter expression in all stem and progenitor cell types of Dox-treated Vav-rtTA3 bitransgenic mice and untreated Vav-tTA bitransgenic reporter mice (Figure 1). However both Vav promoter-driven strains showed a significant proportion of GFP– cells in each progenitor population, suggesting they are less efficient than CAG-rtTA3 and CMV-rtTA for inducible expression in these cell types. Intriguingly, Eμ-tTA transactivator mice drove appreciable GFP expression in HSCs and most progenitor populations despite predicted lymphoid-specific activity (Figure 1).

### Variable Transactivator Function in Differentiated Hematopoietic Cell Types

**Figure 2:**
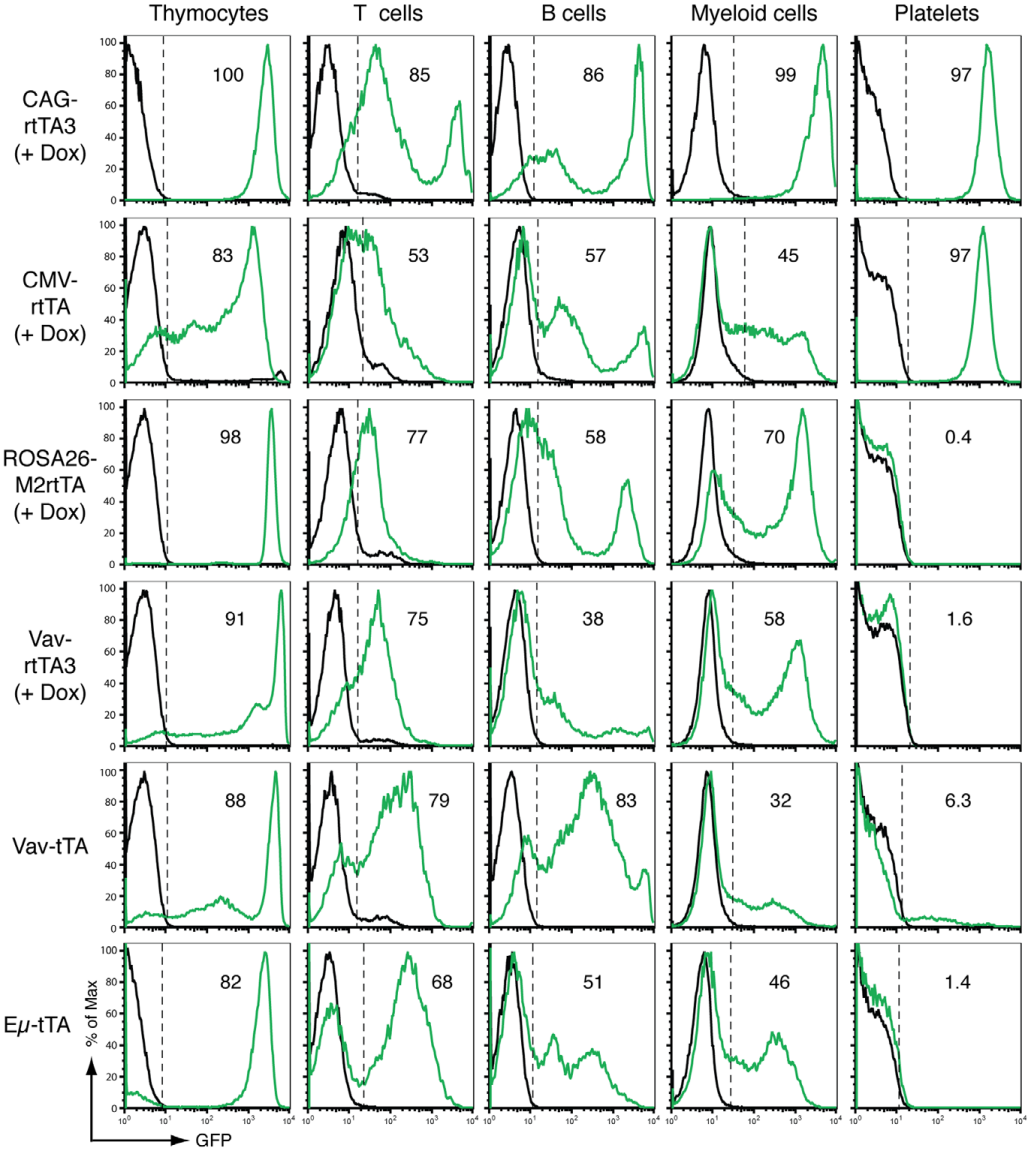
Tet-regulated GFP reporter expression in developing and mature hematopoietic cells of tet-transactivator transgenic mice. Flow cytometry profiles are as described in Figure 1. Thymocytes: CD4+CD8+ thymocytes. T cells: CD3+ splenocytes. B cells: B220+ splenocytes. Myeloid cells: Gr1+Mac1+ bone marrow cells. Platelets: CD41+ peripheral blood (plasma) cells. Profiles are from a representative mouse (n = 2–6 analysed per genotype).

We also examined tet-regulated GFP expression patterns in more differentiated hematopoietic cell populations in the bone marrow, spleen, thymus, and blood. Once again CAG-rtTA3 was the most potent of the three ‘ubiquitous’ tet-on strains across more mature cell types, with 85–100% GFP expression in thymocytes, splenic B cells, bone marrow myeloid cells and blood platelets (Figure 2). Reporter expression in immature CD4+CD8+ (DP) thymocytes was efficient and intense in all strains apart from CMV-rtTA, suggesting that this cell type is particularly amenable to transactivator expression from different promoters. Strong reporter expression in Eμ-tTA DP thymocytes is consistent with a previous report where use of this strain to drive expression of TRE-Myc resulted predominantly in DP T cell lymphoma [22]. Notably, all strains showed a dramatic reduction in the efficiency of reporter expression in more mature, splenic CD8+ T lymphocytes (Figure 2; see below). Reporter expression in splenic B cells was also surprisingly poor in several strains, and only CAG-rtTA3 drove intense reporter expression in a high proportion of this cell population. We found that rtTA3 mRNA expression correlated with GFP expression in splenic B cells of CAG-rtTA3; TRE-GFP-shLuc mice (Figure S3), suggesting that transactivator expression level is an important determinant of reporter expression. Interestingly, GFP expression in splenic B cells of untreated Vav-tTA bitransgenic mice far outweighed that of their Dox-treated Vav-rtTA3 counterparts, potentially due to different Vav promoter activity based on transgene insertion site, transgene copy number, or strain background. CAG-rtTA3 drove intense and efficient GFP expression in bone marrow Gr1+Mac1+ myeloid cells (predominantly neutrophils), a cell population that showed very poor induction in most other strains (Figure 2). The highly efficient reporter expression driven by CAG-rtTA3 in most cell types indicates that the *Col1a1*-targeted reporter transgene is amenable to transactivation across the hematopoietic system, and further implicates transactivator expression level as a critical determinant of reporter expression in different transactivator strains (see Discussion).

### Inducible Reporter Expression in Megakaryocytes/platelets Correlates with Phenotype

**Figure 3:**
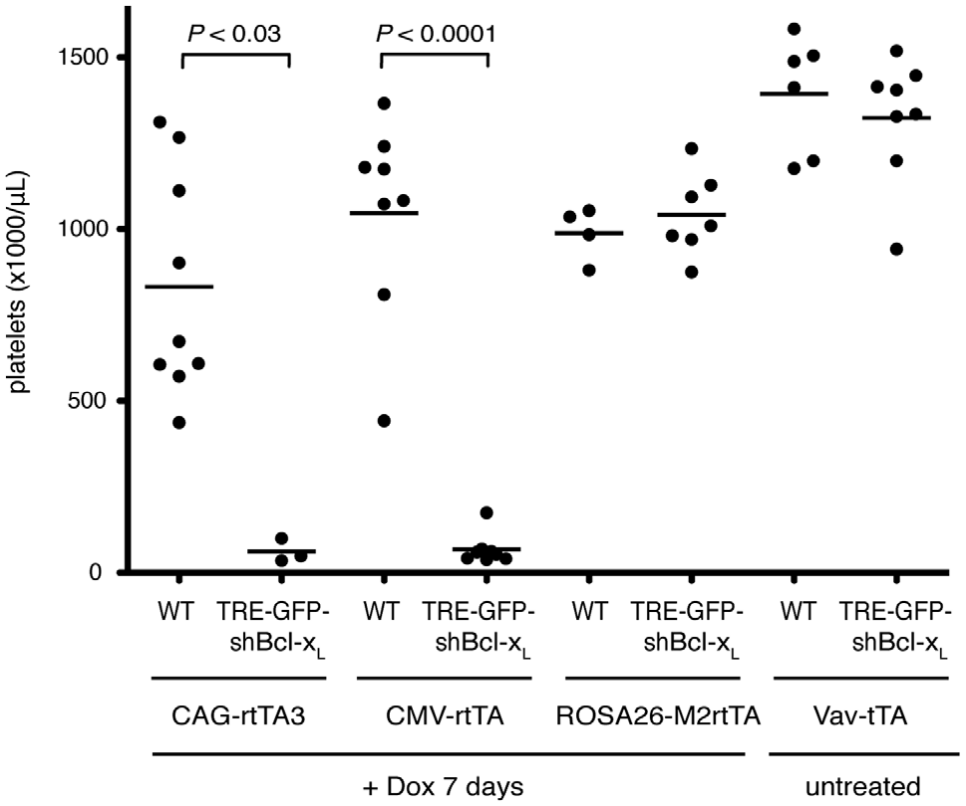
Transactivator-specific thrombocytopenia associated with Bcl-x_L_ knockdown in megakaryocytes/platelets. Peripheral blood platelet counts of ROSA26-M2rtTA; TRE-GFP-shBcl-x_L_, CMV-rtTA; TRE-GFP-shBcl-x_L_, CAG-rtTA3; TRE-GFP-shBcl-x_L_, and Vav-tTA; TRE-GFP-shBcl-x_L_. Mice were either untreated (tet-off mice) or doxycycline treated for one week (tet-on mice) prior to blood sampling. Mice were bled between 6 and 14 weeks of age.

Of all cell types examined, platelets showed the greatest variability in reporter expression between different transactivator strains. Platelets from CAG-rtTA3 and CMV-rtTA bitransgenic reporter mice uniformly expressed high GFP levels, whereas in other strains they were remarkably refractory to tet-regulated expression (Figure 2). Reporter expression in platelets was generally consistent with that in megakaryocyte precursors (Figure 1). To verify that GFP reporter expression accurately reflects transactivator function in platelets, we examined the phenotypic effects of tet-regulated knockdown of an endogenous gene in several transgenic transactivator strains. Bcl-x_L_ (Bcl2l1) is required for maintaining platelet survival in adult mice [25], and we have previously shown that tet-regulated Bcl-x_L_ knockdown in megakaryocytes causes severe thrombocytopenia [11]. We crossed TRE-GFP-shBcl-x_L_ transgenic mice [11] to several transactivator mice. Dox treatment of CAG-rtTA3; TRE-GFP-shBcl-x_L_ mice and CMV-rtTA; TRE-GFP-shBcl-x_L_ mice induced severe thrombocytopenia, with platelet levels falling to less than 10% of those in littermate controls or untreated mice (Figure 3). In contrast, Dox-treated ROSA26-M2rtTA; TRE-GFP-shBcl-x_L_ mice and untreated Vav-tTA; TRE-GFP-shBcl-x_L_ mice maintained normal platelet levels (Figure 3). These observations confirmed a close correlation between reporter expression and functional tet-regulated gene knockdown, and highlight marked differences in the ability of several ‘ubiquitous’ transactivator mouse strains to induce expression and associated phenotypes in a defined cell type.

### Variable Tet-regulated Expression During T cell Differentiation *in vivo*

**Figure 4:**
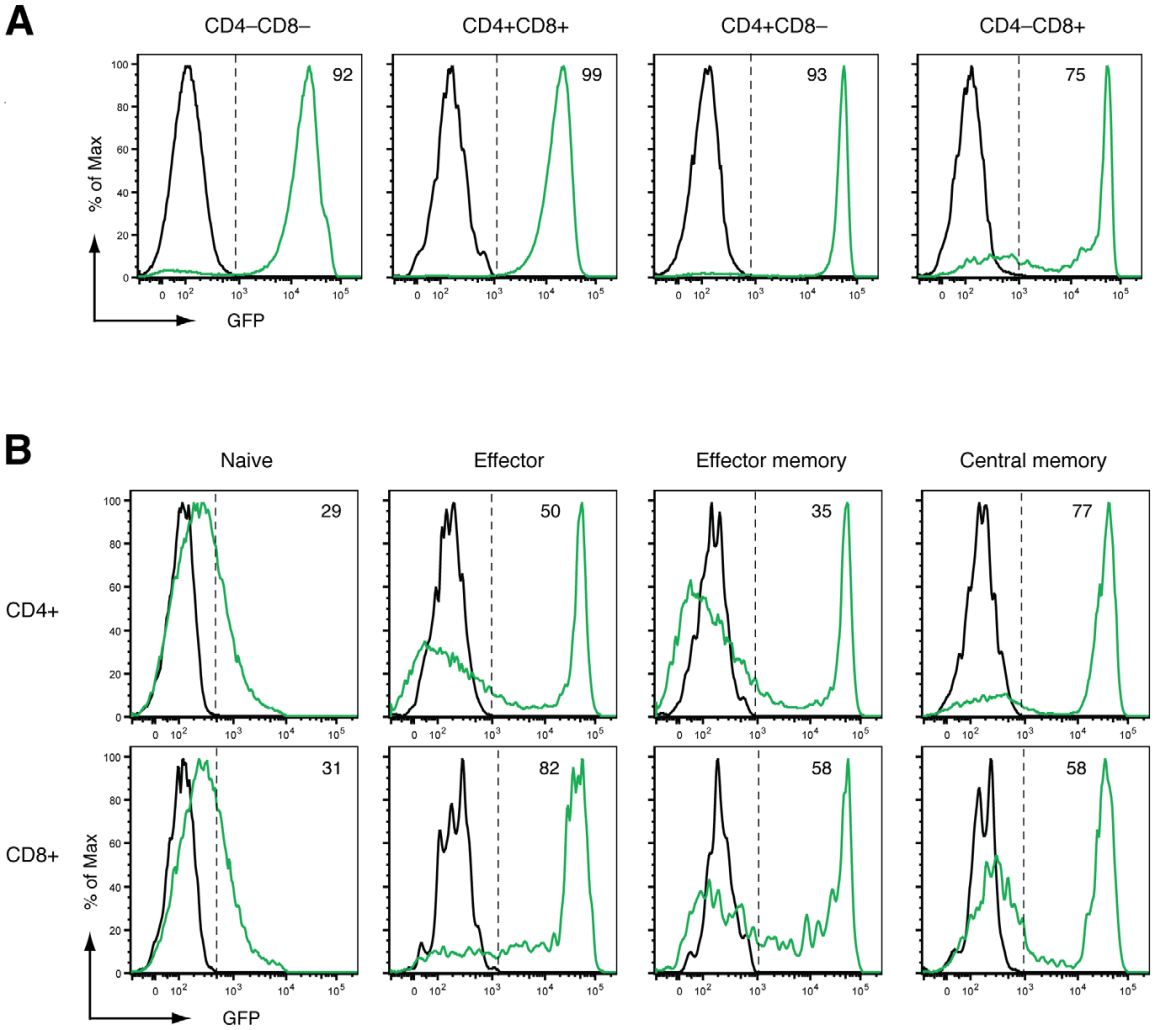
GFP reporter expression in T cell subsets of CAG-rtTA3 mice. Flow cytometry profile of GFP expression in thymic and splenic T cell subsets from a representative CAG-rtTA3 bitransgenic reporter mouse (green) compared with a control mouse (black). Mice were given Dox food for 7 days before analysis. The percentage of GFP+ cells in each population is indicated. (A) Reporter expression during thymocyte differentiation through DN (CD4–CD8–) to DP (CD4+CD8+) to SP (CD4+CD8– and CD4–CD8+) stages. (B) Reporter expression in splenic T cell subsets. Naïve: CD62L+CD44–, Effector: CD62L–CD44+, Effector memory: CD44+CD127+CD62L–, Central memory: CD44+CD127+CD62L+. Gating strategies are shown in Figure S5.

Having noted that reporter expression in several transactivator strains was far more efficient in developing, immature T cells in the thymus relative to mature T cell populations in the spleen (Figure 2), we tracked reporter expression during incremental stages of T cell development in these organs (Figure 4, Figures S4 and S5). The CAG-rtTA3 strain drove efficient and intense reporter expression throughout T cell development in the thymus, transitioning from CD4–CD8– progenitors through to more mature CD4+CD8– and CD4–CD8+ single positive (SP) populations (Figure 4A). In contrast, naïve SP T lymphocytes in the spleen of these mice only showed weak reporter expression (Figure 4B). This may be explained by poor CAG promoter activity and low rtTA3 expression in naïve T cells, however given that all transactivator strains displayed poor reporter expression in this cell type (Figure S4) we cannot rule out inaccessibility of the TRE-GFP-shLuc reporter transgene. Remarkably, high level GFP expression was restored in a large proportion of effector and memory T cells (Figure 4B). As most splenic T cells are naïve, the reporter expression observed in splenic T cell subsets is consistent with a preponderance of GFP-low cells in the total splenic T cell population (Figure 2).

### *In vivo* Kinetics of Tet-on and Tet-off Reporter Expression

**Figure 5:**
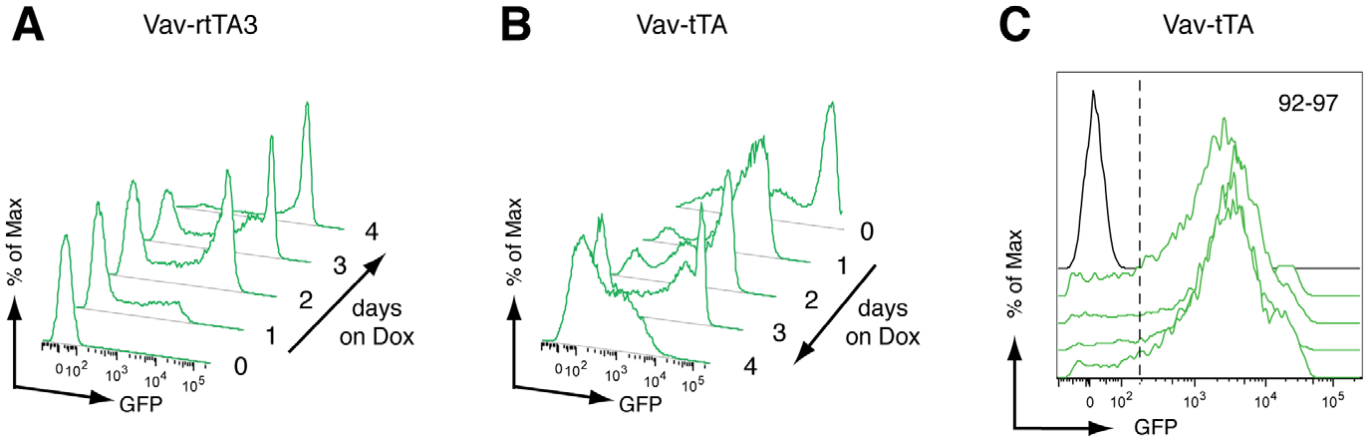
Kinetics and variability of GFP reporter expression. (A, B) Flow cytometry profiles of GFP expression in CD4+CD8+ thymocytes from representative Vav-rtTA3; TRE-GFP-shLuc (A) and Vav-tTA; TRE-GFP-shLuc (B) mice during a time course of Dox treatment. (C) Flow cytometry of GFP expression in peripheral blood B220+ B cells of four different Vav-tTA; TRE-GFP-shLuc mice. The percentage of GFP+ cells ranged from 92–97% as indicated.

A major strength of tet-regulated systems is rapid induction or repression of a protein-coding gene or shRNA. Having demonstrated particularly effective tet-regulated expression in DP thymocytes of Vav promoter-driven tet-on (Vav-rtTA3; TRE-GFP-shLuc) and tet-off (Vav-tTA; TRE-GFP-shLuc) mice (Figure 2), we investigated the *in vivo* kinetics of GFP induction and repression respectively in this cell population upon doxycycline treatment. Time course analysis revealed rapid reporter induction in Vav-rtTA3; TRE-GFP-shLuc mice, with over 30% of DP thymocytes expressing GFP after one day of Dox treatment (Figure 5A). Notably, after only 2 days of treatment approximately 60% of DP thymocytes were GFP+, most of which comprised a distinct GFP-high peak. The proportion of thymocytes expressing GFP reached near-maximum levels (>90%) after four days on Dox (Figure 5A). A similarly high proportion of thymocytes from the corresponding untreated Vav-tTA; TRE-GFP-shLuc tet-off reporter mice expressed high GFP levels, which gradually diminished upon Dox treatment (Figure 5B). Approximately 70% of thymocytes remained GFP+ after 4 days of Dox treatment *in vivo*, albeit with low fluorescence intensity. In principle the half-life of a tet-regulated overexpressed protein (in this case GFP) dictates its rate of decay upon de-induction, and likely contributes to the slower rate of reporter repression relative to induction we observed in thymocytes (Figure 5A and 5B).

### Natural Variation in Tet-regulated Expression

Throughout our analyses we observed appreciable variability in the proportion of GFP+ cells and the pattern of GFP fluorescence intensity between bitransgenic reporter mice matched for sex, age, and genotype. Although in most cases this was minor (Figure 5C), it demonstrates significant variability of transactivator expression or function between individual mice. This variation may be based on stochastic epigenetic effects at the transactivator or tet-responsive transgenes, and may also be influenced by differences in the developmental and immunological history of individual mice.

## Discussion

Inducible gene over-expression or knockdown in mice using the tet-regulated expression system has proven very useful for understanding gene function *in vivo*. Although a multitude of tet transactivator transgenic mice have been developed, in many cases the pattern and level of transactivator expression and resulting TRE-driven protein or shRNA expression are poorly characterised. In this study we have used flow cytometry to systematically measure the tet-regulated expression of a fluorescent protein reporter across the hematopoietic system of several different tet transactivator mouse strains. This provided a unique opportunity to accurately quantitate not only the proportion of a particular cell type with reporter expression, but also expression level per cell based on fluorescence intensity.

A key finding of this study was the degree of heterogeneity in reporter expression in several mouse strains where transactivator expression is under the control of a nominally ubiquitous promoter such as CMV or ROSA26. This heterogeneity occurred at three levels. Firstly, reporter expression often varied markedly between different cell lineages within a transactivator mouse. For example CMV-rtTA reporter mice showed highly efficient GFP induction in the megakaryocyte/platelet lineage, however expression in myeloid and lymphoid cells was poor compared with other strains. Secondly, we observed differences in reporter expression between developmental stages within particular hematopoietic lineages. This was especially evident in the T cell lineage, where in all strains examined there was a dramatic fall in reporter expression in peripheral, naive T cell subsets compared with their immature progenitors in the thymus. In several strains we also noted decreased reporter expression in platelets compared with their megakaryocyte progenitors. These findings clearly caution against extrapolating reporter expression from one differentiation stage to another, and suggest that as progenitor cells proliferate and differentiate down a particular lineage the expression of tet-regulated transgenes can fluctuate significantly. This is an inherent drawback of regulated transcription systems, and contrasts with the ‘hit-and-run’ genetic changes induced using Cre/lox based mouse models. A third and particularly problematic feature of some transactivator mouse strains examined was significant variability in reporter expression between cells within an immunophenotypically defined cell population. In principle this variation can be explained by mosaic transactivator expression and/or accessibility of the TRE response transgene at the *Col1a1* locus. This locus was originally chosen as a transgenic ‘landing pad’ because it supports transgene expression even in cell types that do not normally express Col1a1 [10]. However we note that Col1a1 is expressed at low but uniform levels across the wide range of mouse hematopoietic cell types analysed in the “Immunological Genome Project” (www.immgen.org) [26]. Indeed our results revealed a remarkable, near-100% induction of TRE-reporter expression in most cell types of the CAG-rtTA3 strain, confirming that the *Col1a1* locus is highly amenable to reporter transactivation across the hematopoietic system. This is consistent with our previous observations in other tissues [27]. An exception is naïve, splenic T cells, in which we noted poor reporter expression in all transactivator strains. This raises the possibility of silencing of the *Col1a1*-targeted tet responsive transgene in this cell type, perhaps associated with the high degree of chromatin condensation in naïve T cells relative to activated/memory T cells [28]. Previous retroviral and transgenic studies have also noted low level expression of CMV-based promoters in the T cell lineage [29]–[31].

Our results suggest that sub-optimal transactivator protein expression limits reporter induction in the ‘ubiquitous’ transactivator strains CMV-rtTA and ROSA26-M2rtTA. The activity of transgenic promoters lacking normal regulatory elements can be subject to position effects associated with epigenetic silencing [32]. However ROSA26-M2rtTA is a knockin transgene that makes use of the endogenous ROSA26 promoter, therefore the basis of variable transactivator expression within certain cell types of this strain remains unclear.

Of the six transgenic strains tested, CAG-rtTA3 was the most effective driver of reporter expression across multiple hematopoietic cell types. This was exemplified by high efficiency reporter expression in bone marrow myeloid cells and platelets, cell types refractory to tet-regulated expression in most if not all other strains. Efficient expression across almost all hematopoietic cell types is consistent with our previous observations in non-hematopoietic tissues [9], and establishes the CAG-rtTA3 strain as a highly effective driver of tet-regulated expression across the great majority of cell types examined. However we noted poor CAG-rtTA3-driven reporter expression in MEPs and a significant proportion of splenic B cells, emphasising the need for validation of transactivator function in cell types of interest even within broadly effective transactivator strains.

It is often desirable to restrict tet-regulated expression to the hematopoietic compartment. For example, previous models of oncogene addiction or tumour suppressor hypersensitivity in leukemia and lymphoma have relied on transgenic mice where transactivator expression is controlled by the largely hematopoietic-specific Eµ or Vav promoters [8], [21], [22]. Although our results suggest that Vav-tTA, Vav-rtTA3, and Eµ-tTA transgenic mice display robust tet-regulated reporter expression in many hematopoietic cell types, expression in stem, progenitor, and myeloid cells of these strains was clearly sub-optimal relative to the highly effective CAG-rtTA3 strain. In principle, efficient yet hematopoietic-specific tet-regulation can be achieved by transplanting bone marrow or fetal liver-derived hematopoietic stem cells derived from CAG-rtTA3 or other effective ‘ubiquitous’ promoter-driven transactivator mice into lethally irradiated wild type mice. The resulting chimeric reconstituted mice should allow robust Dox-dependent expression across the hematopoietic system with minimal effects on non-hematopoietic tissues.

While highlighting the potential heterogeneity of tet-regulated expression across the hematopoietic system *in vivo*, our study also emphasises the power of fluorescent reporter flow cytometry for analysis and isolation of cells with optimal tet-regulated inducible expression from a mixed population. For tet-regulated overexpression of a gene of interest, reporter co-expression can be achieved using bicistronic expression cassettes based on internal ribosome entry sites (IRES) or 2A peptides [33]. Similarly, tet-regulated gene knockdown can be tracked by including shRNA sequences in the 3′ UTR of the reporter transcript. The TRE-GFP-shRNA configuration used in this study has been optimised for both GFP expression and target gene knockdown [9].

In summary, we have systematically characterised tet-regulated expression across the hematopoietic system of several transactivator mouse strains. The reporter expression patterns described here allow an informed choice of the most appropriate transactivator transgenic strain for investigation of a certain hematopoietic cell type or process, and provide a basis for the accurate interpretation of hematopoietic phenotypes generated using these strains. Furthermore we have found that CAG-rtTA3 transgenic mice facilitate intense and efficient inducible gene regulation across the majority of blood cell types, establishing this strain as a valuable tool for the study of hematopoietic development and disease.

## Methods

### Ethics Statement

All animal experiments were approved by the Walter and Eliza Hall Institute Animal Ethics Committee.

### Transgenic Mice

Mouse strains described previously include TRE-GFP-shLuc and CAG-rtTA3 [9], TRE-GFP-shBclxL [11], CMV-rtTA [14], ROSA26-M2rtTA [15], Vav-tTA [21], and Eμ-tTA [22]. The Vav-rtTA3 mouse strain was made by cloning rtTA3 coding sequences into the Vav promoter vector HS21/45 [20], which was used for standard pronuclear transgenesis. Several transgenic founder lines were screened in vivo by crossing to the TRE-GFP-shLuc transgenic reporter, and the Vav-rtTA3 strain was chosen based on optimal reporter expression in blood cells. The four tet-on transgenic lines were maintained on a C57BL/6 strain background, and the two tet-off transgenic lines on a FVB/N background. To minimise strain background effects, we analysed F1 progeny derived from mating TRE-GFP-shRNA transgenics with transactivator transgenics. The rtTA transgene was detected using forward (GCTTGGTGTAGAGCAGCCTACAC) and reverse (CAGCGCTGAGTGCATATAACGCG) primers, yielding a 311 bp product. The M2rtTA transgene was detected using forward (ACGGCGCTCTGGAATTACTCAATGG) and reverse (AGAAGCCTTGCTGACACAGGAACGC) primers, yielding a 345 bp product. The rtTA3 transgenes were detected using forward (CTGCTGTCCATTCCTTATTC) and reverse (CGAAACTCTGGTTGACATG) primers, yielding a 303 bp product. The tTA transgene was detected using forward (CCATACTCACTTTTGCCCTTTAG) and reverse (CAGCGCTGAGTGCATATAATGCA) primers, yielding a 221bp product.

TRE-GFP-shRNA transgenes were genotyped as described [11]. The rtTA, M2rtTA, rtTA3, and tTA transgene genotyping protocols are provided in Figures S1, S2, S3, S4, S5. Doxycycline was administered in the diet at 600 mg/kg food (Specialty Feeds, Glen Forrest, Western Australia).

### Flow Cytometry and Blood Analysis

Blood was collected from the retro-orbital plexus and platelet cell counts were measured with an Advia 2120 hematological analyser (Bayer, Leverkusen, Germany). To stain mature hematopoietic cells, single cell suspensions were prepared from bone marrow, thymus, spleen and peripheral blood. Following red blood cell lysis, cells were stained with APC-conjugated anti-B220 (BD553092), anti-CD8 (BD553035), or anti-Gr1 (BD553129), or PE-conjugated anti-CD3 (BD555275), anti-CD4 (BD553049) or anti-Mac1 (BD557397). Cell preparations were also stained with propidium iodide (Sigma-Aldrich, St Louis, MO) and only live cells were displayed. Platelet-rich plasma was prepared by centrifuging blood in phosphate buffered saline at 125×g for 7 minutes, and stained with PE-conjugated anti-CD41 antibody BD558040 (BD Biosciences, San Jose, CA). Stained cells were analysed by flow cytometry (FACSCalibur, BD Biosciences, San Jose, CA). FACS data were analyzed with FlowJo software (Tree Star, Ashland, OR). For staining of CMP, GMP and MEP populations, single cell suspensions from bone marrow were incubated with a collection of biotinylated mature linage markers (anti-CD4, CD8, B220, Ter119, Gr1, Mac1 antibodies) followed by staining with PE-Texas Red-conjugated Streptavidin antibody (BD551487) along with PECy7-conjugated anti-Sca1 (BD558162), PCPCy5.5-conjugated Kit (BD560557), AF647-conjugated CD34 (BD5602330), and PE-conjugated FcgRII/III (BD553145). For staining of MkP, single cell suspensions from bone marrow were incubated with a collection of biotinylated mature linage markers, PECy7-conjugated Sca1, PCPCy5.5-conjugated Kit, PE-conjugated CD41 (BD558040) and APC-conjugated CD150 (Biolegend 115910) followed by staining with PE-Texas Red-conjugated Streptavidin antibody. For staining of CLP, single cell suspensions from bone marrow were stained with a collection of PE-conjugated mature linage markers, PECy7-conjugated Sca1, PCPCy5.5-conjugated Kit and biotinylated CD127 (BD555288) followed by staining with APC-conjugated Streptavidin antibody (BD554067). Cell preparations were also stained with Fluoro-Gold™ (Sigma-Aldrich, St Louis, MO) and only live cells were displayed. Stained cells were analysed by flow cytometry (LSR II, BD Biosciences, San Jose, CA). Gating strategies are shown in Figure S2. For staining of different subsets of T cells in spleen, single cell suspension from spleen was stained with APC-conjugated CD4 (BD561830), Pacific Blue-conjugated CD8 (BD558106), PE-Cy7-conjugated CD62L (BD560516), PE-conjugated CD44 (BD561860) and APC-conjugated CD127 (eBioscience 17-1271-82). Samples were analysed on FACSCanto (BD Biosciences, San Jose, CA). Gating strategies are shown in Figure S5.

### Expression Analysis

RNA was extracted from sorted cells using an RNeasy kit (QIAGEN, Valencia, CA). rtTA3 expression was determined by RT-qPCR (Platinum SYBR Green; Invitrogen, Carlsbad, CA) using forward primer TTACACTGGGCTGCGTATTG and reverse primer AGAAGTGGGGGCATAGAATC.

## Supporting Information

Figure S1 **GFP reporter expression in TRE-GFP-shLuc single transgenic mice.** Flow cytometry profiles of GFP expression in thymocytes (CD4+CD8+ thymocytes), T cells (CD3+ splenocytes), B cells (B220+ splenocytes), and myeloid cells (Gr1+Mac1+ bone marrow cells) from representative TRE-GFP-shLuc single transgenic reporter mice (untreated shown in red, 7 day Dox treated shown in green). Wild type control is shown in black.
(TIF)

Figure S2 **Gating strategy for hematopoietic stem cells and early progenitors.** Adapted from [34].
(TIF)

Figure S3 **Transactivator expression in GFP– and GFP+ cell populations.** RT-qPCR analysis of rtTA3 expression in GFP– and GFP+ B cells (B220+) sorted from the spleen of a representative CAG-rtTA3; TRE-GFP-shLuc mouse, compared with non-transgenic control.
(TIF)

Figure S4 **GFP reporter expression in T cell subsets of CMV-rtTA and ROSA26-M2rtTA mice.** Flow cytometry profiles of GFP expression in thymic and splenic T cell subsets from representative CMV-rtTA or ROSA26-M2rtTA bitransgenic reporter mice (green) compared with a control mice (black). Mice were given Dox food for 7 days before analysis. The percentage of GFP+ cells in each population is indicated. (A) Reporter expression during thymocyte differentiation through DN (CD4–CD8–) to DP (CD4+CD8+) to SP (CD4+CD8– and CD4–CD8+) stages. (B) Reporter expression in splenic T cell subsets. Naïve: CD62L+CD44–, Effector: CD62L–CD44+, Effector memory: CD44+CD127+CD62L–, Central memory: CD44+CD127+CD62L+. Gating strategies are shown in Figure S5.
(TIF)

Figure S5 **Gating strategy for thymic and splenic T cell subsets.**
(TIF)
