# Supplementary figures and images for: Variability of Inducible Expression across the Hematopoietic System of Tetracycline Transactivator Transgenic Mice
Source: PLoS One. 2013 Jan 11;8(1):e54009. doi: 10.1371/journal.pone.0054009 (PMC3543435; doi:10.1371/journal.pone.0054009)

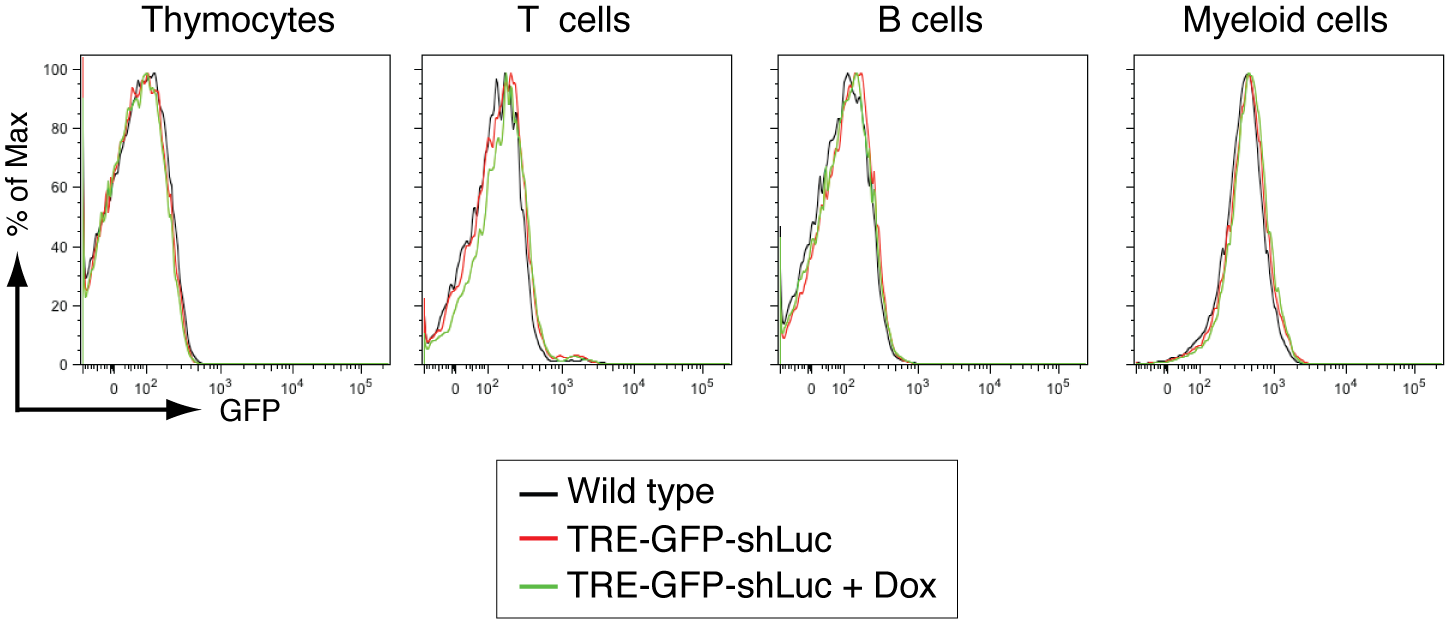

Supplement: Figure S1 — GFP reporter expression in TRE-GFP-shLuc single transgenic mice. Flow cytometry profiles of GFP expression in thymocytes (CD4+CD8+ thymocytes), T cells (CD3+ splenocytes), B cells (B220+ splenocytes), and myeloid cells (Gr1+Mac1+ bone marrow cells) from representative TRE-GFP-shLuc single transgenic reporter mice (untreated shown in red, 7 day Dox treated shown in green). Wild type control is shown in black. (TIF) [file pone.0054009.s001.tif]

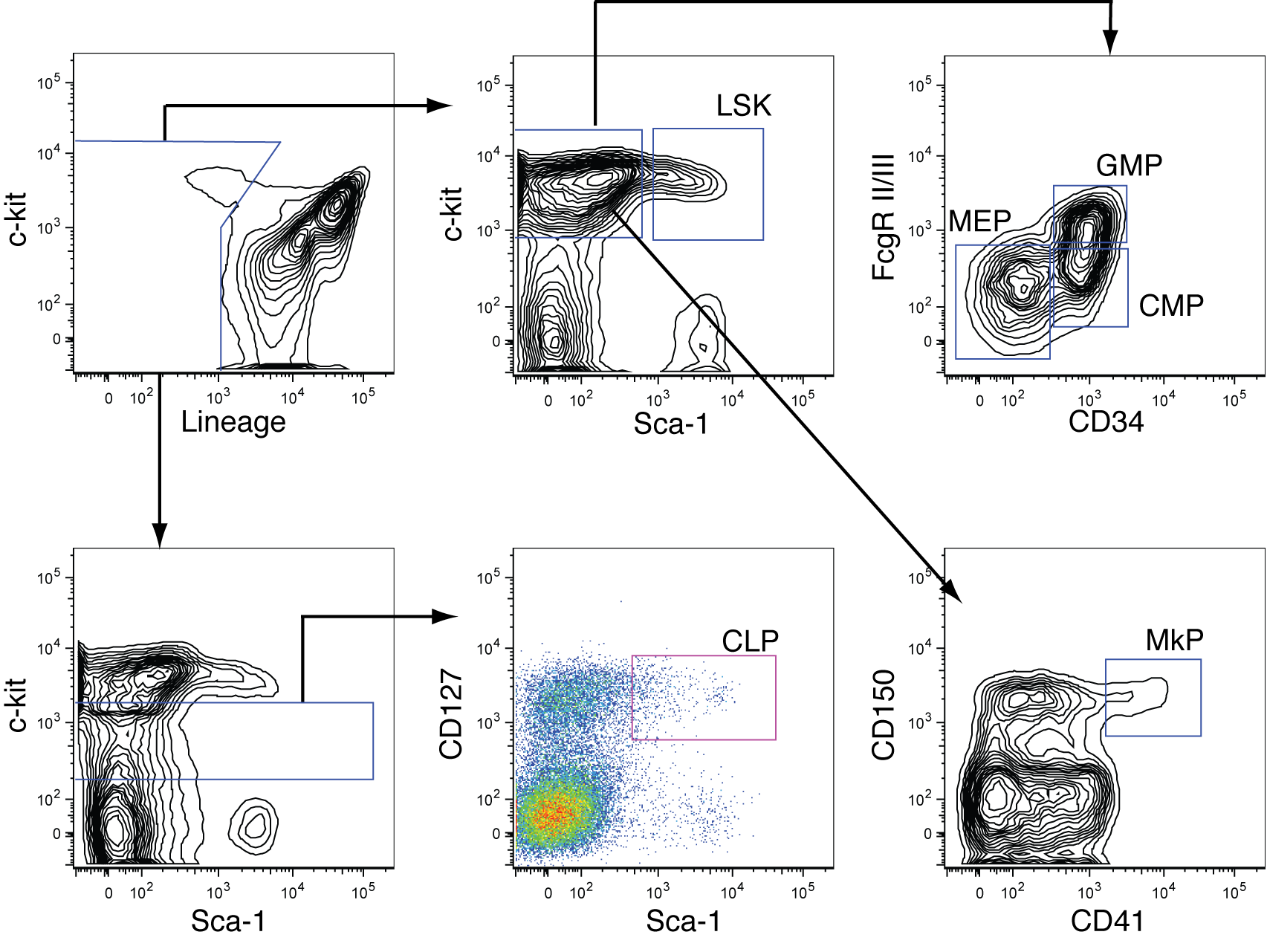

Supplement: Figure S2 — Gating strategy for hematopoietic stem cells and early progenitors. Adapted from [34]. (TIF) [file pone.0054009.s002.tif]

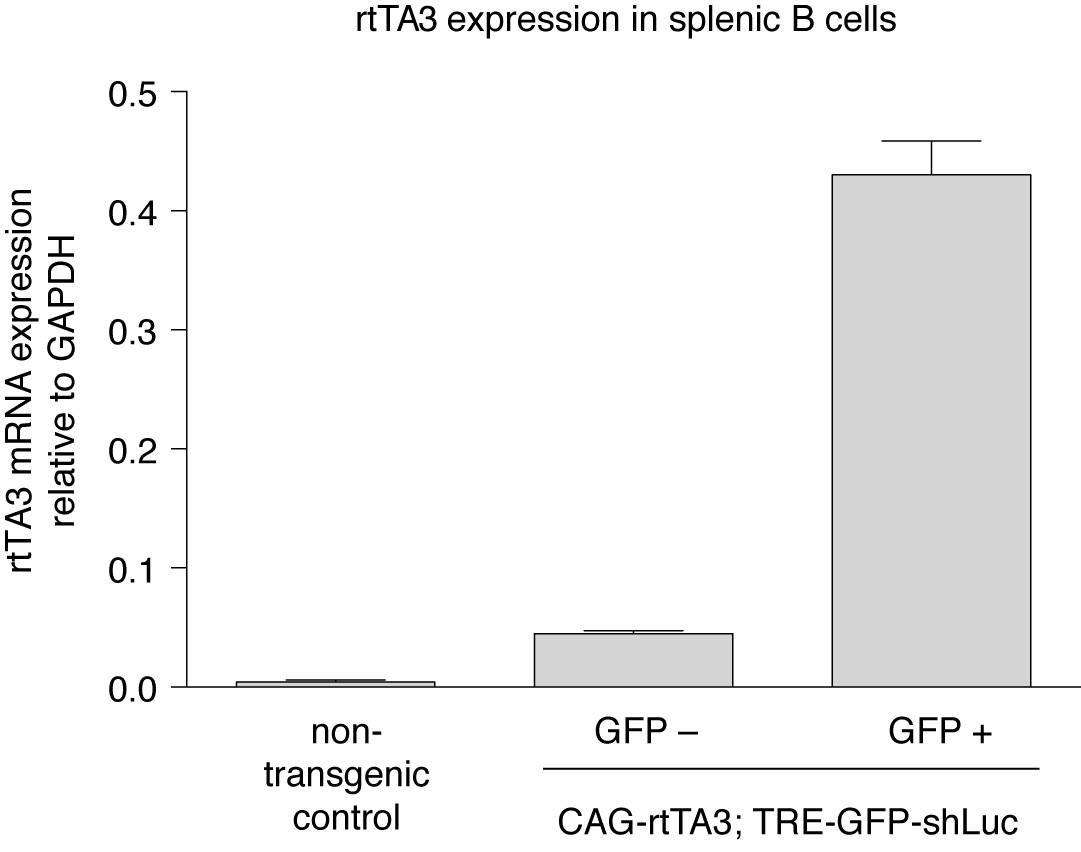

Supplement: Figure S3 — Transactivator expression in GFP– and GFP+ cell populations. RT-qPCR analysis of rtTA3 expression in GFP– and GFP+ B cells (B220+) sorted from the spleen of a representative CAG-rtTA3; TRE-GFP-shLuc mouse, compared with non-transgenic control. (TIF) [file pone.0054009.s003.tif]

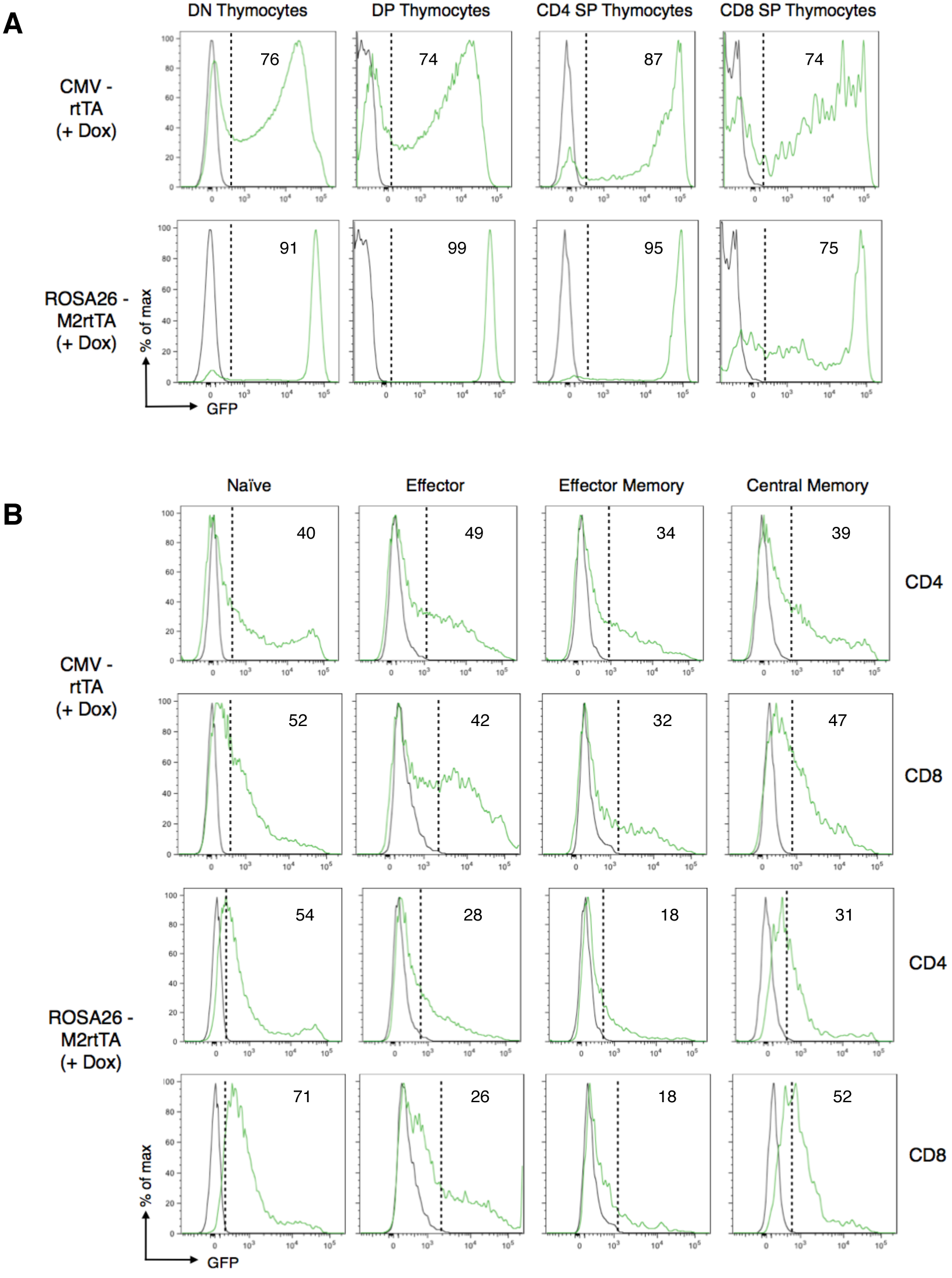

Supplement: Figure S4 — GFP reporter expression in T cell subsets of CMV-rtTA and ROSA26-M2rtTA mice. Flow cytometry profiles of GFP expression in thymic and splenic T cell subsets from representative CMV-rtTA or ROSA26-M2rtTA bitransgenic reporter mice (green) compared with a control mice (black). Mice were given Dox food for 7 days before analysis. The percentage of GFP+ cells in each population is indicated. (A) Reporter expression during thymocyte differentiation through DN (CD4–CD8–) to DP (CD4+CD8+) to SP (CD4+CD8– and CD4–CD8+) stages. (B) Reporter expression in splenic T cell subsets. Naïve: CD62L+CD44–, Effector: CD62L–CD44+, Effector memory: CD44+CD127+CD62L–, Central memory: CD44+CD127+CD62L+. Gating strategies are shown in Figure S5. (TIF) [file pone.0054009.s004.tif]

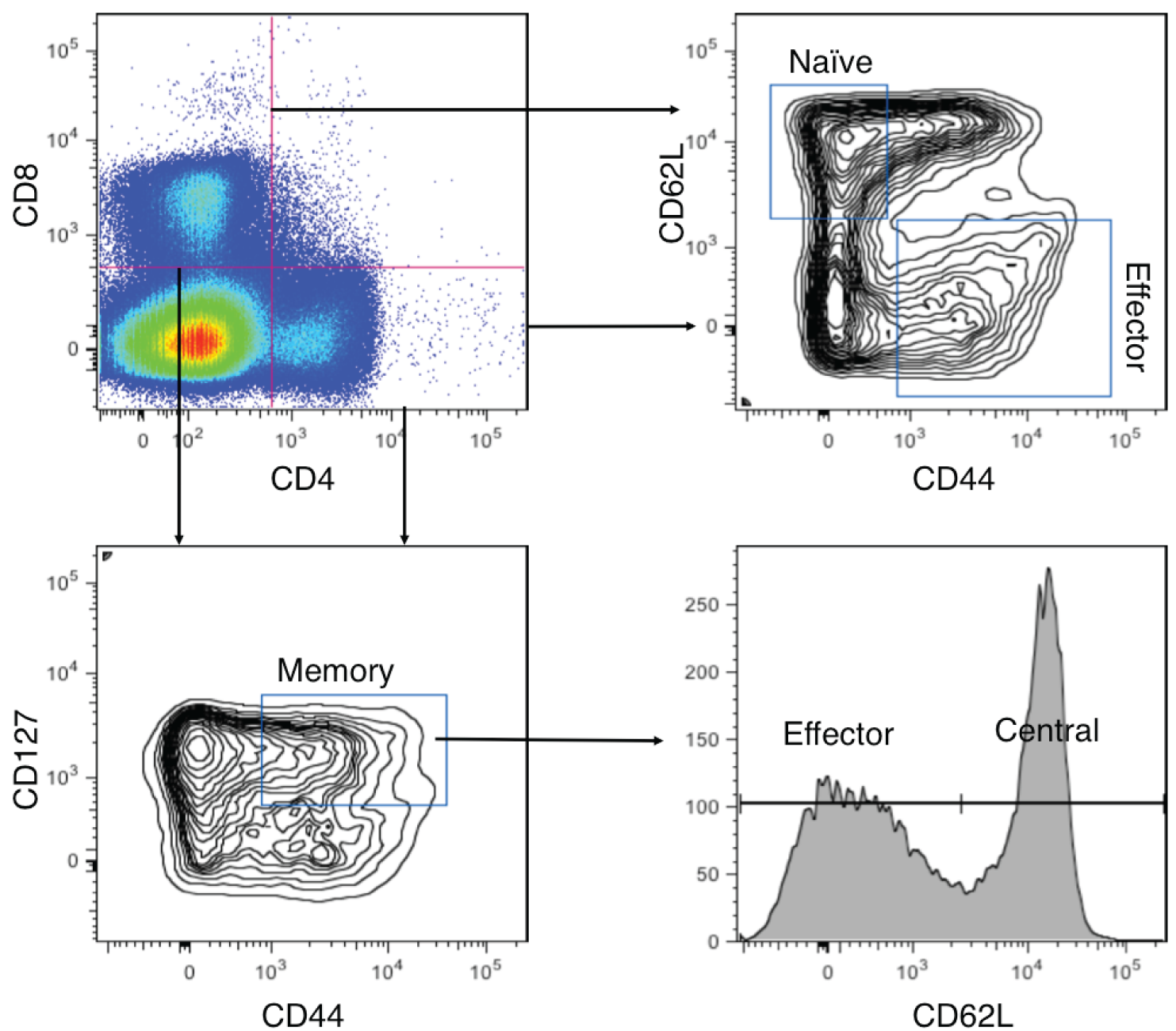

Supplement: Figure S5 — Gating strategy for thymic and splenic T cell subsets. (TIF) [file pone.0054009.s005.tif]
